# Early-life local labor market conditions and old-age male mortality: evidence from deindustrialization of New England textile sector

**DOI:** 10.1017/dem.2026.10021

**Published:** 2026-05-14

**Authors:** Hamid Noghanibehambari, Jason Fletcher

**Affiliations:** 1 College of Business, Austin Peay State University, USA; 2 La Follette School of Public Affairs, University of Wisconsin Madison, USA

**Keywords:** mortality, longevity, deindustrialization, local labor market, early-life exposures, migration

## Abstract

Previous studies document links between early-life exposures and life-cycle outcomes, but fewer examine how local labor market shocks during early life affect old-age male mortality. This article studies this relationship using a large-scale deindustrialization shock: the decline of New England’s textile industry during the 1920s and 1930s. Consistent with prior work, we find small effects on migration and limited changes in counties’ sociodemographic composition after deindustrialization. Using Social Security Administration death records linked to the 1900–1940 historical censuses and difference-in-differences event studies, we find reduced longevity among men born in highly exposed counties, especially among non-migrant families and those in non-urban areas. The estimates imply intent-to-treat effects of about 3.6 months, while treatment-on-the-treated calculations suggest longevity reductions of about 4.2 years for children of affected families. Using 1950–1960 census data, we also find reductions in schooling, high school completion, and socioeconomic standing.

## 1. Introduction

A growing body of empirical studies examines the sources of health disparities in old age and provides evidence that exposures in utero, during early life, and in early childhood significantly influence variations in old-age health. (Almond *et al.*, 2018; Almond and Currie, 2011; Barker, 1994, 1995, 1997). This literature shows that disruptions during critical developmental periods can permanently alter health capital formation and skill accumulation, generating persistent effects throughout the life cycle (Cunha and Heckman, 2007). The path dependent nature of health and human capital formation, in the absence of policy interventions, may appear in adverse outcomes during old age.

One important determinant of early-life health environments is parental economic opportunity and local labor market conditions. Adverse economic conditions can reduce household resources available for nutrition, housing quality, and healthcare access^1^, thereby affecting children’s biological and human capital development (Rosenzweig and Schultz, 1983). Empirical studies document that negative labor market shocks and business-cycle downturns during early life are associated with poorer infant health, lower educational attainment, and worse adult health outcomes. A growing strand of work further links early-life economic conditions to long-run mortality and longevity, showing that exposure to recessions, unemployment shocks, or economic crises during gestation and childhood can have lasting consequences for survival in old age (Lindeboom *et al.*, 2010; Van Den Berg *et al.*, 2006).

This paper joins this literature by evaluating the effects of local labor market conditions during early life on old-age longevity. Specifically, we focus on a large-scale deindustrialization that took place in the textile sector in New England region during the early decades of the 20^th^ century. Textile manufacturing was a highly prosperous sector in New England during the 19^th^ and early 20^th^ century, covering about 15 percent of the total labor force by 1920. However, starting from 1920, the industry faced large contractions in size. From 1920 to 1940, the industry shed about 7 percentage-points of its labor force. Several factors contributed to the overall decline in the region’s textile industry, including outdated machinery of factories combined with a slower pace of adaptation, increased foreign competition and outsourcing from European countries with relatively cheaper labor, changes in demand size and preferences post World War I, and specifically increased competition with southern states who had more abundant raw materials and cheaper labor.

We exploit variations in the timing and location of this deindustrialization to examine the effects of being born under adverse economic conditions on old-age longevity. In so doing, we employ death records data from Social Security Administration linked with the full-count 1940 census. We use cross-census linking rules to establish a link between historical censuses 1900–1930 and 1940 in order to infer individuals’ county-of-birth. This information is essential to evaluate the effects of local conditions. We implement two-way fixed effect models and event studies that compares outcomes of individuals born in different pre-and-post deindustrialization years in localities with higher versus lower initial reliance on textile industries in their labor force. We find null effects among the general population. However, we find relatively larger effects among people living in non-urban areas and those who do not migrate from their county-of-birth in years leading to 1940. Among these non-urban non-migrants of high exposure counties, we find a reduction of about 3.6 months in longevity. We provide empirical evidence that the exposure to deindustrialization does not result in changes in population composition based on several observable individual and family characteristics. Moreover, we find considerably larger impacts among those with low-educated fathers and low socioeconomic status families. Further, we use 1950 and 1960 censuses to evaluate potential mechanisms. We find that individuals born in higher exposure counties reveal significant reductions in education and their measures of occupational standing and occupational education score.

The current study is motivated by and adds to two strands of empirical research on the health effects of economic shocks. First, since the New England textile market fall was partly driven by cross-state and international import competition, this study relates to recent line of research that examine the health impacts of trade liberalization and increases in import competition. For instance, Pierce and Schott (2020) show that localities in the US with higher exposure to trade liberalization with China experienced increases in adult mortality rate. Fan *et al.* (2020) use data from China and show that trade expansions post-2000 caused increased working hours and reduced self-reported health measures among working age population. However, Feng *et al.* (2021) show that export expansion resulted in increases in earnings among Chinese workers and improved their general health. Fernández Guerrico (2021) employs data from Mexico and show that localities with higher reductions in manufacturing employment after trade liberalization with China experienced increased mortality rates and obesity. Lang *et al.* (2019) show that individuals in localities with higher exposure to import completion from China reveal reductions in mental and physical health. Noghanibehambari (2025) exploits the variations in local exposure to the North American Free Trade Agreement (NAFTA) to examine the effects of local labor market conditions on birth outcomes in the US. He finds significant negative impacts on a wide array of infants’ health. Olper *et al.* (2018) employs cross-country data over the years 1960–2010 and show that trade liberalization reduced child mortality rates. Our study joins this literature and evaluates long-term impacts of such trade-induced shocks on longevity and mortality outcomes.

Second, the current study also relates to the limited empirical evidence of early-life economic conditions and later-life old-age longevity, specifically in the case of the US. Cutler *et al.* (2007) examine the effects of early-life exposure to the Dust Bowl and the Great Depression on later-life health using census-region-level macroeconomic data as a proxy for local economic conditions. They find no significant effects on a wide range of health outcomes including mortality. Schmitz and Duque (2022) explore the effects of local unemployment during the Great Depression on later-life outcomes using birth-state variations in wage index as a proxy for general local economic conditions. They employ Health and Retirement Study (HRS) and examine the effects on epigenetic aging signature, a predictor of mortality risks. They find significant associations that are localized for in-utero exposures as opposed to pre-conception and childhood exposures. Noghanibehambari *et al.* (2024) use historical county-level bank deposits data to proxy for local economic conditions. They employ Social Security death records linked with the full-count 1940 census and show significant association between per capita deposits at the county-year-of-birth and later-life longevity. Van Den Berg *et al.* (2006) examine this association using historical data in Netherlands using national business cycle as a proxy for local economic conditions. They find that being born during an economic boom (versus a recession) is associated with about 1.6 years longer lives. Lindeboom *et al.* (2010) show that cohorts born during the potato famine of the mid-19^th^ century in Netherlands lived between 2.5–4 years shorter lives during adulthood.

Our paper contributes to the literature in three ways. First, although the decline in New England’s textile industry was partly caused by a post-war decline in labor demand, cross-state competition and import competition were also important factors. Therefore, our paper can also contribute to the recently developed and ongoing literature and policy debates regarding the effects of international trade competition (Autor *et al.*, 2019; Autor *et al.*, 2013; Batistich and Bond, 2023; Hakobyan and McLaren, 2016, 2017; Pierce and Schott, 2020). This literature usually finds that individuals residing in localities with higher reliance in industries that are affected by trade policy changes or labor demand shocks reveal reductions in job prospects, earnings, and experience adverse health outcomes. Our paper adds to this literature and documents a heterogenous impact on long-run mortality for a deindustrialization that is partly trade-induced. In addition, there were fewer social insurance during the period of study to insulate the negative effects of worsening economic conditions (Modrek *et al.*, 2022; Noghanibehambari and Engelman, 2022). Although limited small-scaled unemployment insurance existed specifically for the New Deal period of post-1930, they were not designed or targeted based on industry-specific job losses. Second, we provide evidence of a large-scale deindustrialization with differential impacts among localities which lends to the exogeneity assumption of our method. This is in contrast with previous works that directly test for the associations using regional-level, state-level, or county-level proxies for economic conditions (Cutler *et al.*, 2007; Noghanibehambari *et al.*, 2024; Schmitz and Duque, 2022). Our event study design allows for a flexible cross-cohort change in the effects which are detached from any time-varying proxies that are arguably contaminated with other determinants of health and later-life longevity. Moreover, we show that the effects are driven by specific subpopulations, i.e., non-urban and non-migrant families. This fact suggests that barriers to migration and cross-occupation movements might play a role, which should be the target of policies to alleviate the long-run consequences of deindustrialization. Third, we also contribute to the literature on early-life exposures and later-life outcomes, specifically mortality (Aizer *et al.*, 2016a; Almond, 2006; Fletcher, 2018; Fletcher *et al.*, 2010; Hayward and Gorman, 2004; Montez and Hayward, 2011, 2014). We focus on longevity for two reasons. First, age at death is an objective and precisely measured outcome in administrative data, avoiding concerns related to reporting error, differential diagnosis, or subjective self-assessments that may affect measures such as self-reported health, disability status, or functional limitations. Second, it is a summary measure of general health and is correlated with a wide range of other individual measures, including human capital and economic success (Buchman *et al.*, 2012; Chetty *et al.*, 2016; Lubitz *et al.*, 2003; Mathers *et al.*, 2001).

While a large body of work documents the long-term effects of in utero and early-life conditions on later-life health and mortality, the robustness and persistence of these effects remain debated. For instance, Almond (2006) compares cohorts born in 1919 (in utero during the 1918 influenza pandemic) with adjacent cohorts and finds reductions in education and increases in disability later in life. However, Beach *et al.* (2022a) highlight the challenges of isolating causal early-life impacts and suggest that the effects reported in Almond’s paper become smaller once we account for family background, given that the 1919 cohorts came from systematically lower socioeconomic status families. We acknowledge these ongoing debates and interpret our findings with appropriate caution. In this context, the relatively small effect sizes observed in our analysis are consistent with the view that early-life economic shocks may influence long-term outcomes, but also note that the magnitude of these effects might reveal significant heterogeneity across subpopulations based on sociodemographic characteristics, migration status, and urban status.

## 2. Historical background

New England’s abundant water ways and rivers provided unique geographic advantages to exploit hydraulic power necessary to run early textile mills in the late 18^th^ and early 19^th^ century. Therefore, it became a center for textile production in the US. During the 19^th^ century, the industry was successful in incorporating new technological advancements, such as the spinning jenny and power loom to expand production. The primary focus of the region was producing coarse textiles, such as cotton, wool, and flax.

This hub of industrial powerhouses started to contract, specifically during the 1920s and 1930s. The New England states (i.e., Rhode Island, Maine, Connecticut, Massachusetts, New Hampshire, and Vermont) experienced large-scale plant closures in textile industries (Choi, 2023). Between 1920 and 1940, textile industry of the region reduced about 7 percentage-points employment of the labor force, off an initial baseline of 15 percent.

Several factors contributed to this large-scale deindustrialization. First, expansions in manufacturing industries in countries with cheaper labor, such as Germany and Japan, during the early 20^th^ century and specifically after World War I increased import competition. Second, southern US states started expansions in the textile industry during this period. Availability of raw materials, e.g., cotton, and lower costs of labor combined with better climate for cotton production resulted in more competitive prices relative to productions from New England (Koistinen, 2002). Third, during this period, there was a change in consumer preference towards lighter and more fashionable products, such as synthetic fibers, while New England textile focused on more coarse products, such as wool (Rosenbloom, 1999). Fourth, in the face of demand reductions and import competition, factories started to lower the costs which reflected in labor benefits. Labor unions started labor strikes to raise the pay. This labor unrest contributed to the general production decline in the industry (Greenlees, 2019).

Manufacturers in New England’s textile industries started political lobbying for ‘retrenchment’ policies (to cut local and state taxes to lower production costs) and ‘federal intervention’ acts (to absorb federal aids to save the region’s industry). Both set of actions resulted in limited effects (Koistinen, 2000). Although several firms could relocate to Southern states to continue production, many others went bankrupt and shut down (Koistinen, 2002). However, there is evidence that other manufacturing sectors and jobs in public sectors grew in size which mitigated the negative effects on local economies (Rosenbloom, 1999). Choi (2023) uses historical census data and show that individuals in highly affected towns in New England states were less likely to migrate, switched to agricultural sector, and faced decreases in their occupational scores.

## 3. Empirical method

Our empirical method rests on the assumption that individuals in localities with higher versus lower exposure reveal similar trends in the outcomes in the absence of industrial decline. Later in the paper, we employ a series of event-studies and balancing tests to lend to the exogeneity assumption of our method. The econometric method employs a difference-in-difference strategy that compares longevity of cohorts who were born in counties at different terciles of 1900 textile share of labor force (first difference) and born after 1920 (as the starting year of deindustrialization) versus those born before (second difference). Specifically, we utilize this comparison using the following ordinary least square regressions:
(1)

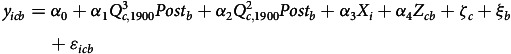




Where the outcome is age-at-death of individual 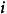 who was born in county 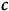 and year 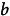 . Dummy variables 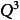 and 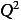 indicate whether birth-county 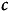 is at the third and second tercile of share of textile employment in the labor force in 1900, respectively. In 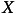 , we include individual and family controls. Individual controls include dummies for race and ethnicity. Family controls include dummies for maternal education, paternal literacy, and paternal socioeconomic index.^2^ The matrix 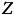 , includes a series of county covariates that are extracted from the full-count censuses 1880–1940 and interpolated for inter-decennial years. These county controls include average population, the share of population in different age groups, share of population in different race groups, share of immigrants, share of married individuals, average family size, and average occupational income score. County fixed effects ( 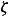 ) absorbs all time-invariant county features that influence later-life health and mortality. Birth cohort fixed effects ( 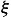 ) absorb secular changes in cohorts’ health and life expectancy that evolves over time. Finally, 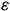 is a disturbance term. We cluster standard errors by birth-county and birth-year to account for both spatial and serial correlation in error terms.

To show the effects across different birth cohorts, we employ event-studies of the following form:
(2)

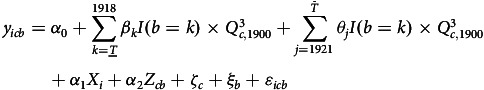




The set of coefficients 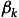 represents pre-trend coefficients which capture the difference in longevity of individuals in high exposure counties in years prior to 1920s. Since the main results point to significant and large effects for the top-tercile counties, we define high exposure as counties in top-terciles of 1900 textile employment share in the labor force ( 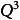 ). The parameters 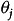 capture the effects on longevity of individuals in high exposure counties in years after 1920. We group event-time coefficients into two-year increments, removing the coefficients of 1919–1920 cohorts to set them as the reference group. All other parameters are as in equation 1.

## 4. Data and sample selection

We employ Social Security Administration Death Master Files (DMF) and Numerical Identification (Numident) records as the primary source of data extracted from the CenSoc Project (Goldstein *et al.*, 2021). The DMF data reports deaths to male individuals between the years 1975–2005 and Numident data reports deaths that occurred between 1988–2005 to both males and females. There are three advantages in using DMF-Numident data. First, the CenSoc-extracts of DMF-Numident provides links at the individual level to the full-count 1940 census.^3^ It allows us to observe individual and family characteristics as observed in 1940. Second, we can use the cross-census linking rules to locate individuals in historical censuses and deduce their county-of-birth, an extremely scarce identifier. Third, the DMF-Numident death sample contains an initial sample of millions of observations which leaves room to have restrictions to specific cohorts and geographic regions and still have enough observations with sufficient statistical power. These aspects of the data make it superior to many alternative datasets to study mortality outcomes, such as National Longitudinal Mortality Study or HRS.^4^

The 1940 census and other full-count historical censuses are extracted from IPUMS Project (Ruggles *et al.*, 2020). We use cross-census linking rules provided by the Census Linking Project (Abramitzky *et al.*, 2020).^5^ This cross-census linking is essential for two reasons. First, since the focus is local county-level economic conditions, we need to have a reliable proxy for county-of-birth. Second, the industrial decline, as we will argue later, increased migration, specifically for non-urban populations. Therefore, we also need to build measures of migration from birth-county to the 1940 county. To deduce the birth county, we split the sample into 11–year cohort bins. We then use the first census they could appear as their “original census”. For instance, for cohorts of 1900–1910, the original census is 1910 census. We link 1940 census to 1910 census and use the county-of-residence in 1910 as county-of-birth. This procedure goes on until 1930. For cohort of 1930–1935, we use “county-of-residence in 1935” information as reported in the 1940 census. For cohorts of 1936–1940, we use county-of-residence in 1940 as county-of-birth.

**Figure 1.**
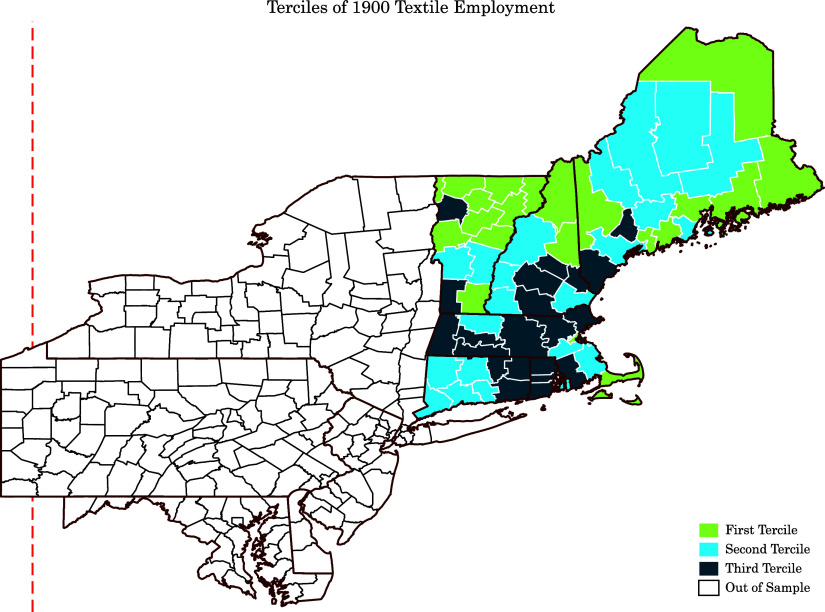
Geographic distribution of counties based on 1900 share of textile employment in the labor force.

Since linking between historical censuses is based on names and women change their names over time, the linking rules are for males only. Therefore, the main sample restriction is that we focus on male mortality in this paper. We restrict the sample to cohorts of 1900–1940. Moreover, as the industrial decline was restricted to New England, we focus on individuals who were original born in New England states (Rhode Island, Maine, Connecticut, Massachusetts, New Hampshire, and Vermont). Figure 1 shows the geographic distribution of counties in the final sample based on their 1900 share of textile employment in the labor force.

Summary statistics of the final sample are reported in Table 1. The average age-at-death in the first, second, and third tercile of textile production is 75.5, 75.6, and 75.6 years, respectively. While the majority of the final sample consists of whites; there is a slightly higher share of whites and lower share of blacks in high textile counties. There are fewer migrants and higher share of urban residents in high textile counties.

**Table 1.** Summary statistics

	First tercile of 1900 textile		Second tercile of 1900 textile		Third tercile of 1900 textile	
	Mean	SD	Mean	SD	Mean	SD
Death age (month)	905.68	117.89	907.73	116.69	907.27	117.37
Death age (years)	75.1	9.79	75.14	9.73	74.97	9.83
Birth year	1,919	10	1,918	10	1,918	10
Death year	1,994	8	1,994	7	1,994	8
White	0.99	0.11	0.99	0.09	0.99	0.08
Black	0.01	0.10	0.01	0.09	0.01	0.08
Non-migrant	0.35	0.48	0.40	0.49	0.40	0.49
Nonurban	0.43	0.49	0.48	0.50	0.34	0.48
Father literate	0.94	0.23	0.95	0.22	0.94	0.23
Father literate missing	0.01	0.09	0.00	0.06	0.01	0.07
Mother literate	0.94	0.24	0.93	0.25	0.93	0.26
Mother literate missing	0.01	0.10	0.00	0.05	0.00	0.07
Father socioeconomic index 1^st^ quartile	0.34	0.47	0.26	0.44	0.23	0.42
Father socioeconomic index 2^nd^ quartile	0.14	0.35	0.18	0.39	0.22	0.41
Father socioeconomic index 3^rd^ quartile	0.18	0.39	0.20	0.40	0.19	0.39
Father socioeconomic index 4^th^ quartile	0.19	0.39	0.21	0.41	0.21	0.41
Father socioeconomic index missing	0.14	0.35	0.15	0.35	0.15	0.36
Mother education < high school	0.36	0.48	0.39	0.49	0.42	0.49
Mother education high school	0.22	0.42	0.21	0.41	0.19	0.39
Mother education college	0.03	0.16	0.03	0.18	0.03	0.17
Mother education missing	0.39	0.49	0.36	0.48	0.36	0.48
*County covariates:*						
Share of textile employment	0.02	0.01	0.06	0.03	0.22	0.10
Population	429,592	384,490	221,672	143,734	400,790	249,857
Share of population aged 0–4	0.10	0.02	0.10	0.01	0.10	0.01
Share of population aged 5–10	0.11	0.02	0.11	0.01	0.11	0.01
Share of population aged 11–18	0.14	0.02	0.14	0.01	0.14	0.01
Share of population aged 19–25	0.12	0.01	0.12	0.01	0.12	0.01
Share of population aged 26–55	0.40	0.04	0.41	0.02	0.41	0.01
Share of population aged 56–more	0.13	0.04	0.13	0.03	0.12	0.02
Share of females	0.50	0.01	0.50	0.01	0.51	0.01
Share of families with children < 5	0.39	0.10	0.37	0.05	0.37	0.04
Share of Whites	0.99	0.01	0.99	0.01	0.99	0.01
Share of Blacks	0.01	0.01	0.01	0.01	0.01	0.01
Share of first-generation immigrants	0.24	0.11	0.22	0.07	0.27	0.05
Share of second-generation immigrants	0.31	0.11	0.31	0.09	0.36	0.06
Share of literate people	0.89	0.18	0.90	0.17	0.89	0.17
Share of married	0.56	0.05	0.58	0.03	0.56	0.02
Average family size	4.25	0.39	4.16	0.19	4.34	0.16
Average occupation income score	24.36	3.33	25.73	2.05	26.34	1.37
Observations	177,233		320824		524208	

It is important to note that the individual and family covariates used in our analysis are drawn from multiple census sources, as no single census contains all relevant variables for the full sample. Specifically, parental literacy and paternal socioeconomic index are obtained from the earliest historical census in which each individual is observed (i.e., the 1910, 1920, or 1930 census, depending on the birth cohort), while maternal education indicators are drawn from the 1940 census, which was the first to record educational attainment. For cohorts born between 1930 and 1940, all parental variables are based on the 1940 census. Parental literacy is fairly similar across different textile-exposure counties. The dummy variables regarding maternal education are extracted from family characteristics in 1940 census as 1940 was the first census to report educational outcomes. Therefore, they are more representative of more recent cohorts since earlier cohorts are more likely to have moved out of their original households. We observe slightly higher share of low-educated mothers in top terciles of textile counties. Finally, based on county-level covariates, higher baseline textile employment counties have slightly higher occupational income scores.

## 5. Results

### 1. Effects on individual migration

We start our analysis by examining the effects on the likelihood of individuals’ migration. We use the full sample and equation 1 in which the outcome of interest is a dummy indicating if the person’ birth-county is different than county-of-residence in 1940. The results are reported in column 1 of Table 2. For the full sample, we find statistically significant increases in the likelihood of migration among those born in counties in the second and third terciles of 1900 textile employment, with estimated effects of 1.4 and 1.8 percentage points, respectively, off a mean of 0.612. When disaggregated by urban status, these effects are concentrated among non-urban individuals: those born in the second tercile are 1.7 percentage points more likely to migrate, while those in the third tercile are 1.3 percentage points more likely to migrate, both significant at conventional levels. Among urban populations, we find smaller and marginally significant effects.

**Table 2.** The effects of exposure to deindustrialization on migration

	Outcome: migrant, subsamples:		
	Full sample	Non-urban	Urban
	(1)	(2)	(3)
3^rd^ Tercile of 1900 Textile 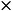 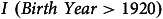	0.018***	0.013*	0.01*
	(0.005)	(0.008)	(0.006)
2^nd^ Tercile of 1900 Textile 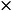 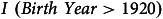	0.014***	0.017***	0.002
	(0.005)	(0.007)	(0.006)
Observations	1022265	408981	613284
R-squared	0.11	0.134	0.11
Mean DV	0.612	0.592	0.625
County FE	✓	✓	✓
Birth year FE	✓	✓	✓
Controls	✓	✓	✓

*Notes*: Standard errors, two-way clustered on county and birth-year, are in parentheses. Controls include individual, family, and county covariates. Individual controls include dummies for race and ethnicity. Family controls include dummies for maternal education, paternal literacy, and paternal socioeconomic index. County controls include average population, the share of population in different age groups, share of population in different race groups, share of immigrants, share of married individuals, average family size, and average occupational income score.

*** *p* < 0.01, * *p* < 0.1.

These findings align broadly with the results reported in Choi (2023), who also documents increased out-migration from textile-dependent areas in response to deindustrialization. However, there are several important differences with the findings documented here. First, Choi (2023) examines variation in textile employment shares across towns in Massachusetts, whereas our analysis is conducted at the county level and includes a broader set of New England states. Second, she uses individual-level census data from 1850 to 1940 to study labor market dynamics and demographic responses, whereas our primary focus is on birth cohorts exposed in early life to textile decline and their long-run mortality outcomes. Third, while both studies find increased migration, our results indicate that the migration response was stronger among non-urban populations, suggesting that geographic and infrastructural isolation may have shaped mobility decisions differently across space.

To better understand the characteristics associated with migration behavior, we further examine the correlations between migration status and individual or family sociodemographic characteristics. Specifically, we regress these characteristics on a dummy variable indicating non-migrant status. The results are presented in Appendix Table A-6. Although most coefficients are statistically significant, their magnitudes are economically small, suggesting modest compositional differences between migrants and non-migrants. For instance, the coefficient on White implies that non-migrants are approximately 0.2 percentage points more likely to be white, relative to a mean of 0.992. In contrast, the coefficient of –0.014 for Female indicates that non-migrants are 1.4 percentage points less likely to be female, compared with a mean female share of 0.282, suggesting slightly greater male representation among those who remained in their birth counties. Additionally, non-migrants are less likely to have literate fathers. The estimated coefficient implies a decline of about 1.8% with respect to the mean of the outcome.

To assess the potential role of such selection in explaining our main results, we refer to studies examining the effects of parental education on children’s longevity. For example, Noghanibehambari and Fletcher (2024) employ grandfather fixed effects using data similar to the current study and find that a father’s middle school, high school, and college education is associated with increases in children’s longevity of roughly 1.9, 2.8, and 4.4 months, respectively. Combining these estimates with the coefficient in column (4) of Appendix Table A-6 suggests that selection based on paternal education could account for changes in longevity of only about 0.03–0.08 months. As discussed in Section 5.3, the main treatment effects are several orders of magnitude larger than this, indicating that potential selection into migration based on parental characteristics is minimal in our setting.

We replicate these analyses by jointly considering non-migrant and non-urban status. The results, reported in Appendix Table A-7, show a very similar pattern and comparable coefficient magnitudes, reinforcing that selection based on observable characteristics is limited.^6^

### 2. Effects on longevity

The main results of equation 1 are reported in column 1 of Table 3. We observe negative but small and insignificant coefficients. Among non-migrants, the coefficients considerably rise in magnitude although remain statistically insignificant. Among migrants, the top-tercile exposure coefficient becomes positive, suggesting some benefits for longevity. This is in line with several studies that suggest improvements in outcomes for those who migrate during times of economic difficulties (Chyn, 2018; Derenoncourt, 2022; Ludwig *et al.*, 2013). In columns 4 and 5, we examine the heterogenous impacts among nonurban (i.e., rural) versus urban-born individuals. We find a sharp rise in the negative effects among non-urban-born people. The third-tercile county effect is now statistically significant, suggesting an average of 2 months reduction in longevity.

**Table 3.** The effects of deindustrialization in local labor market at birth year on old-age longevity

	Outcome: age at death (months), subsamples:					
	Full-sample	Non-migrants	Migrants	Non-urban	Urban	Non-urban non-migrants
	(1)	(2)	(3)	(4)	(5)	(6)
3^rd^ Tercile of 1900 Textile 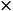 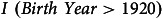	−0.03	−1.43	1.05	−2.01*	1.12	−3.6**
	(0.65)	(0.99)	(0.84)	(1.05)	(0.88)	(1.68)
2^nd^ Tercile of 1900 Textile 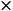 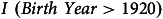	−0.31	−0.51	−0.15	−1.19	0.15	−0.4
	(0.64)	(1.01)	(0.83)	(0.99)	(0.9)	(1.61)
Observations	1022265	397100	625165	408981	613284	166920
R-squared	0.51	0.45	0.54	0.52	0.5	0.46
Mean DV	907.140	906.997	907.231	905.316	908.357	905.200
County FE	✓	✓	✓	✓	✓	✓
Birth year FE	✓	✓	✓	✓	✓	✓
Controls	✓	✓	✓	✓	✓	✓

*Notes*: Standard errors, two-way clustered on county and birth-year, are in parentheses. Controls include individual, family, and county covariates. Individual controls include dummies for race and ethnicity. Family controls include dummies for maternal education, paternal literacy, and paternal socioeconomic index. County controls include average population, the share of population in different age groups, share of population in different race groups, share of immigrants, share of married individuals, average family size, and average occupational income score.

** *p* < 0.05, * *p* < 0.1.

Columns 2 and 4 suggest much larger impacts among non-urban non-migrant people. In column 6, we focus on this subsample. We find that those born in top-tercile of 1900 textile counties reveal 3.6 months reductions in longevity. The effect on those at the second tercile is close to zero and statistically insignificant.

The observed decline in longevity concentrated within the non-urban non-migrant sub-sample may arise from two sources. During this period, New England towns were more dependent on textile manufacturing and therefore faced fewer alternative employment opportunities (Candee, 1982; Galenson, 1975; Koistinen, 2002). This was exacerbated by the weaker institutional capacity of these towns to mitigate economic shocks. Additionally, individuals who did not migrate from these towns might have experienced persistent and continued disadvantage in economically distressed locations. They were not only exposed to lasting adverse economic shocks, but also to the potential deterioration of public infrastructure and lower investment in health and education. Therefore, constrained mobility compounded the adverse effects of early-life economic shocks and manifested in later-life health and longevity.

**Figure 2.**
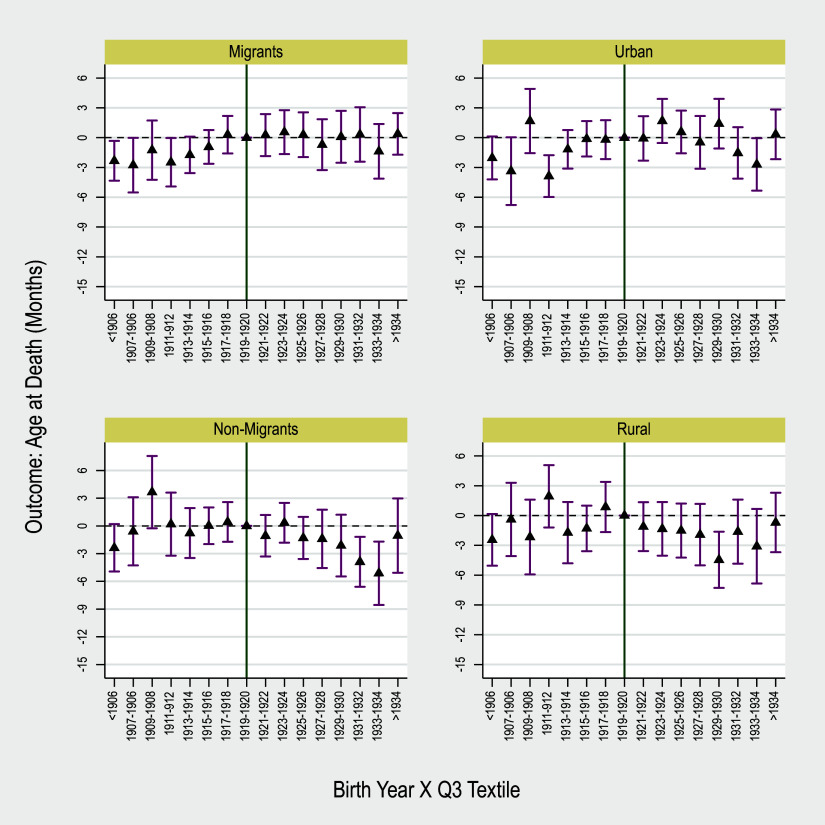
Event study to examine the effects of early-life exposure to deindustrialization across birth cohorts and subpopulations on old-age longevity. *Notes*: Point estimates and 90 percent standard errors are depicted. Standard errors are two-way clustered on county and birth-year. Regressions include county fixed effects, birth year fixed effects, and controls. Controls include individual and county covariates. Individual controls include dummies for race and ethnicity. County controls include average population, the share of population in different age groups, share of population in different race groups, share of immigrants, share of parried individuals, average family size, and average occupational income score.

We complement this table by examining the effects across birth cohorts using event-study estimates of equation 2. The results are depicted in four panels of Figure 2. Among the four subpopulations, we do not observe a significant and robust pattern of pre-trend. We observe drops in post-trend coefficients among non-migrants and those born in rural areas (bottom panels).

**Figure 3.**
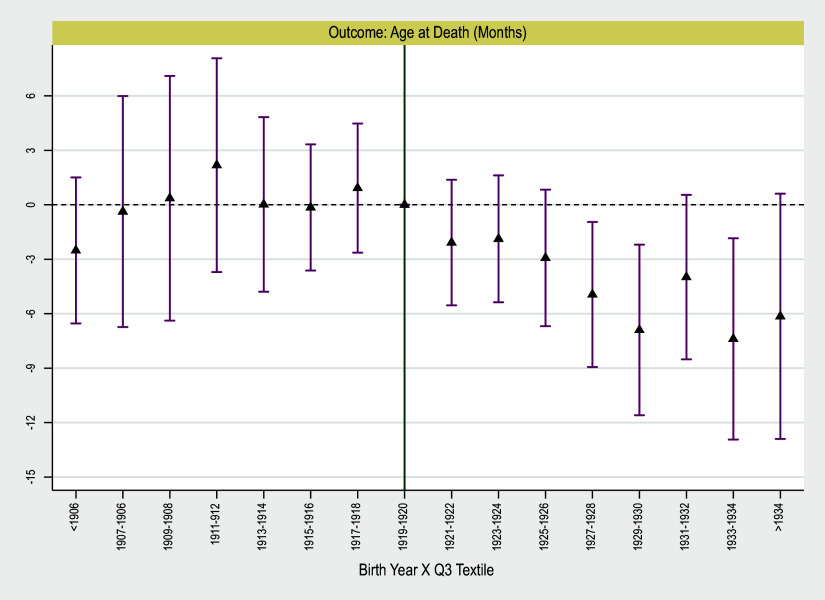
Event study to examine the effects of early-life exposure to deindustrialization across birth cohorts of non-urban non-migrant population on old-age longevity. *Notes*: Point estimates and 90 percent standard errors are depicted. Standard errors are two-way clustered on county and birth-year. Regressions include county fixed effects, birth year fixed effects, and controls. Controls include individual and county covariates. Individual controls include dummies for race and ethnicity. County controls include average population, the share of population in different age groups, share of population in different race groups, share of immigrants, share of parried individuals, average family size, and average occupational income score.

In Figure 3, we focus on the subsample of non-urban non-migrants and replicate the event studies. From 1900–1920, there is no significant evidence of a difference in longevity for those in high exposure versus low exposure counties. However, post-trend coefficients reveal a sharp drop among those born in high exposure counties relative to those in low exposure counties. The effects lasted until 1940 and there is no sign of revival.^7^

The fact that we observe negative and significant effects of high exposed counties among non-migrants and non-urban residents suggests that the negative impacts are primarily concentrated among people and places with fewer alternative job prospects. However, we should note that these effects are intent-to-treat (ITT) which measure the potential impacts among all people in the data. Therefore, we expect to observe larger effects as we focus on the subpopulations who are more likely to be affected. For instance, there is evidence that low educated individuals are less likely to switch occupations (Sicherman and Galor, 1990). There is also evidence of heterogenous impacts of early-life shocks on later-life outcomes by parental education and family socioeconomic status (Almond *et al.*, 2018; Currie, 2009). We examine these heterogeneities in Table 4. In column 1 and 2, we show the effects among children with illiterate and literate fathers, respectively. We find about 14.1 and 12.5 months reductions in longevity for the illiterate-father subsample in third and second tercile counties, respectively. Although these estimates are statistically insignificant due to very small sample size, they point to much larger impacts relative to literate-fathers subsample.

**Table 4.** Exploring heterogeneity across subsamples of non-migrant non-urban population

	Outcome: age at death (months), subsamples:					
	Father illiterate	Father literate	Father low socioeconomic index	Father high socioeconomic index	Father’s occupation: textile	Father’s occupation: non-textile
	(1)	(2)	(3)	(4)	(5)	(6)
3^rd^ Tercile of 1900 Textile 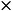 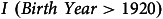	−14.06*	−3.33*	−6.1***	0.07	−11.19*	−3.56*
	(8.26)	(1.73)	(2.23)	(2.59)	(6.39)	(1.82)
2^nd^ Tercile of 1900 Textile 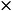 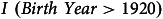	−12.47	−0.04	−1.81	1.99	−8.16	−0.08
	(8.52)	(1.64)	(2.03)	(2.5)	(6.27)	(1.72)
Observations	9791	157126	85170	81749	21255	145662
R-squared	0.39	0.47	0.49	0.43	0.37	0.47
Mean DV	904.619	905.234	910.752	899.412	902.914	905.529
County FE	✓	✓	✓	✓	✓	✓
Birth year FE	✓	✓	✓	✓	✓	✓
Controls	✓	✓	✓	✓	✓	✓

*Notes*: Standard errors, two-way clustered on county and birth-year, are in parentheses. Controls include individual, family, and county covariates. Individual controls include dummies for race and ethnicity. Family controls include dummies for maternal education, paternal literacy, and paternal socioeconomic index. County controls include average population, the share of population in different age groups, share of population in different race groups, share of immigrants, share of married individuals, average family size, and average occupational income score.

*** *p* < 0.01, * *p* < 0.1.

Further, we observe a reduction of about 6.1 months in longevity of those with low-socioeconomic index fathers in top-tercile counties while this effect is almost zero for those with high-socioeconomic index fathers (column 3 versus 4). In column 5 and 6, we ignore occupational mobility post-deindustrialization and explore the effects among those whose fathers’ occupation is in textile and non-textile industries, respectively. We infer father’s occupation using historical censuses 1900–1930. However, for those observations that are not matched to historical censuses, we use father’s occupation (if available) in 1940. We observe considerably larger effects among those with fathers working in textile sector, though the small sample sizes limit statistical power.

We note that, in principle, the linked historical censuses could be used to directly identify individuals whose fathers were employed in the textile industry as the primary measure of exposure. However, the match rate between the 1940 census and earlier censuses is limited and the share of fathers in textile occupations is small; when further restricting to our key subsamples—particularly rural non-migrants—the resulting sample sizes are too small for precise estimation, which motivates our reliance on county-level textile employment share as the baseline exposure measure.

### 3. Cultural norms and parental altruism

A further consideration in the main results is the role of self-selection into migration shaped by parental altruism and family norms. Industrial areas (such as New England textile towns) might be the residential destination for families whose preference over children’s time, schooling, and work differed from other populations. Evidence from late-19th-century U.S. industrial families shows that non-altruistic (or weakly altruistic) parental behavior was pervasive, with child labor common even among asset-owning households, suggesting that choices reflected family preferences and norms, not merely borrowing constraints (Parsons and Goldin, 1989). In theory, intergenerational altruism can itself evolve endogenously with economic conditions, becoming stronger when growth and prospective returns to children’s human capital are high and weakening when prospects deteriorate (Rapoport and Vidal, 2007). These ideas imply that the families who stayed in textile towns after plant closures may have had systematically different intergenerational altruistic preferences from those who migrated out of these towns. If stayers were relatively more tolerant of child work or more present-oriented about schooling, their descendants could display lower propensities to invest in human capital, out-migrate to better locations, or shift occupations, consistent with our finding of negative effects on their later-life longevity.

## 6. Robustness checks

### 1. Balancing tests

One concern in interpreting the results is the endogenous survival of individuals into death records which changes the composition of the final sample based on characteristics that are correlated with their early-life exposure to the deindustrialization. For instance, if there are more individuals with higher educated parents in the final sample who are also more exposed to the industry decline, then our results probably underestimate the true effects as parental education is positively associated with later-life longevity (Huebener, 2019; Noghanibehambari and Fletcher, 2024). This endogenous selection cannot simply be captured by including parental education controls as there are also unobservable factors associated with the influence of education on health. We can empirically test this issue by regressing a series of observable characteristics on the exposure measures introduced in equation 1, conditional on county and birth-year fixed effects. The results of this exercise are reported in Table 5. We do not observe any significant associations with the likelihood of being white, black, other races, and Hispanic. We further do not find statistical association between father being literate and exposure measures (column 5), although we observe some correlations with an indicator of missing information for father literacy.

**Table 5.** Balancing tests of non-urban non-migrant sample

	Outcomes:							
	White	Black	Other	Hispanic	Father Literate	Father Literate Missing	Mother Literate	Mother Literate Missing
	(1)	(2)	(3)	(4)	(5)	(6)	(7)	(8)
3^rd^ Tercile of 1900 Textile 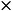 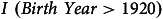	0.00161	−0.00086	−0.00075	0.00095	0.00125	0.00654***	0.01594***	0.00549***
	(0.00147)	(0.00071)	(0.00132)	(0.00093)	(0.00441)	(0.0015)	(0.004)	(0.00149)
2^nd^ Tercile of 1900 Textile 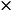 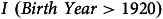	−0.00076	−0.00069	0.00145	0.0001	0.00529	0.00138	0.01675***	0.00104
	(0.00153)	(0.00071)	(0.00138)	(0.00096)	(0.00413)	(0.00089)	(0.00382)	(0.00093)
Observations	166920	166920	166920	166920	166920	166920	166920	166920
R-squared	0.02733	0.02328	0.02354	0.00485	0.03028	0.01719	0.03078	0.0158
Mean DV	0.994	0.001	0.005	0.003	0.941	0.004	0.934	0.004
County FE	✓	✓	✓	✓	✓	✓	✓	✓
Birth year FE	✓	✓	✓	✓	✓	✓	✓	✓
Controls	✓	✓	✓	✓	✓	✓	✓	✓

*Notes*: Standard errors, two-way clustered on county and birth-year, are in parentheses. Controls include individual, family, and county covariates. Individual controls include dummies for race and ethnicity. Family controls include dummies for maternal education, paternal literacy, and paternal socioeconomic index. County controls include average population, the share of population in different age groups, share of population in different race groups, share of immigrants, share of married individuals, average family size, and average occupational income score.

*** *p* < 0.01.

We also observe small but statistical associations with the likelihood of mother being literate. However, there are three reasons that these estimates are not concerning. First, the implied change with respect to the outcome means are very small. Second, there is no clear pattern of effects consistent across outcomes. Third, to the extent that maternal education increases longevity, these results suggest underestimations in negative deindustrialization-longevity associations. Finally, since we do not observe a consistent and robust pattern of associations with observable characteristics, we can rule out the correlations with unobservable features (Altonji *et al.*, 2005; J. Fletcher *et al.*, 2021).

### 2. Endogenous merging

Another concern in interpreting the main results is the selection into the final sample based on Numident-DMF links to the census and cross-census linking rules. The data linking could be endogenous if it is correlated with other determinants of longevity. For instance, nonwhites, immigrants, and people of lower socioeconomic status (who have, on average, lower longevity) are usually underrepresented in the linked samples due to higher errors in enumerations. If the successful linking is correlated with our exposure measures, then the estimates partly reflect this endogenous compositional change. We empirically investigate this concern by examining the associations between successful merging and the exposure measures as in equation 1. In so doing, we use the original male cohorts of 1900–1940 in New England who are categorized as non-urban non-migrants using information in 1940.^8^ We then merge this sample with the final sample of non-migrant non-urban individuals and generate a dummy variable indicating successful merging. We then regress this dummy variable on the right-hand side variables of equation 1. The results are reported in Table 6. We observe small and insignificant associations between exposure measures and successful merging in the full sample (column 1), sample of low-educated mothers (column 2), and non-homeowners as a proxy for wealth (column 3). These tests rule out the concerns of endogenous merging across censuses and Numident-DMF death records.

**Table 6.** Exploring the association between exposure to deindustrialization and census-final-sample successful merging

	Outcome: successful merging between original cohorts of 1940 census and final sample, subsamples:		
	Full sample	Mother education < high school	Family non- homeowner
	(1)	(2)	(3)
3^rd^ Tercile of 1900 Textile 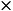 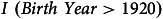	0.00877	−0.00653	0.01111
	(0.00975)	(0.00899)	(0.01365)
2^nd^ Tercile of 1900 Textile 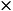 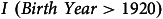	0.00308	0.0023	0.00036
	(0.00747)	(0.00886)	(0.01089)
Observations	535274	190914	153845
R-squared	0.09226	0.04262	0.07681
Mean DV	0.247	0.316	0.243
County FE	✓	✓	✓
Birth year FE	✓	✓	✓
Controls	✓	✓	✓

*Notes*: Standard errors, two-way clustered on county and birth-year, are in parentheses. Controls include individual, family, and county covariates. Individual controls include dummies for race and ethnicity. Family controls include dummies for maternal education, paternal literacy, and paternal socioeconomic index. County controls include average population, the share of population in different age groups, share of population in different race groups, share of immigrants, share of married individuals, average family size, and average occupational income score.

### 3. Alternative specifications

In Table 7, we further examine the robustness of the results to alternative specifications and functional forms. For comparison, we report the results of column 6 of Table 3 in the first column. In column 2, we add birth-month and death-month fixed effects to account for the influence of seasonality of birth and death month in determining longevity (Seretakis *et al.*, 1997; Vaiserman, 2021). In column 3, we interact county fixed effects with individual race dummies and parental education and socioeconomic status dummies to allow for time-invariant effects of counties vary for different subpopulations. In both columns, we observe quite robust estimates.

**Table 7.** Robustness checks

	Column 6 Table 3	Adding birth-month and death-month FE	Interacting county FE with covariate dummies	Outcome: log age at death	Outcome: log age at death, method: Accelerated Failure Time (AFT)
	(1)	(2)	(3)	(4)	(5)
3^rd^ Tercile of 1900 Textile 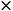 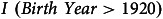	−3.597**	−3.5652**	−3.578**	−0.004**	−0.004**
	(1.6827)	(1.6841)	(1.6911)	(0.0019)	(0.002)
2^nd^ Tercile of 1900 Textile 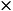 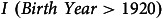	−0.4029	−0.3732	−0.5587	−0.0004	−0.0004
	(1.6095)	(1.6091)	(1.6163)	(0.0019)	(0.001)
Observations	166920	166920	166912	166920	166920
R-squared	0.463	0.4641	0.464	0.4407	--
Mean DV	905.200	905.200	905.202	6.800	905.200
	Outcome: age at death > 65	Outcome: age at death > 75	Weighting by CenSoc weights	Clustering SE on county	Restricting to birth cohorts < 1930
	(6)	(7)	(8)	(9)	(10)
3^rd^ Tercile of 1900 Textile 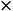 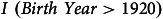	−0.0119*	−0.0181**	−3.3671**	−3.597**	−3.5347**
	(0.0067)	(0.0089)	(1.5613)	(1.5204)	(1.7621)
2^nd^ Tercile of 1900 Textile 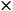 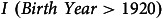	−0.0088	−0.0103	−0.4643	−0.4029	−0.4316
	(0.0063)	(0.0082)	(1.502)	(1.4131)	(1.6863)
Observations	166920	166920	143145	166920	151322
R-squared	0.1727	0.3413	0.4522	0.463	0.3964
Mean DV	0.853	0.480	938.906	905.200	918.336

*Notes*: Standard errors, two-way clustered on county and birth-year (except for column 9), are in parentheses. All regressions include controls include individual, family, and county covariates. Individual controls include dummies for race and ethnicity. Family controls include dummies for maternal education, paternal literacy, and paternal socioeconomic index. County controls include average population, the share of population in different age groups, share of population in different race groups, share of immigrants, share of married individuals, average family size, and average occupational income score.

** *p* < 0.05, * *p* < 0.1.

In columns 4 and 5, we replace the outcome with log of age at death. Column 4 replicates the ordinary least square and column 5 impalements an Accelerated Failure Time (AFT) model to examine the robustness (Aizer *et al.*, 2016a). We observe almost identical coefficients in both columns. High exposure is associated with 0.4 percent reduction in longevity. Using the mean age-at-death of top-tercile counties from Table 1, this effect is equivalent to about 3.6 months, which is similar to that of column 1. In columns 6–7, we replace the outcome with dummy variables indicating survival beyond 65 and 75 years. We observe similar pattern as column 1. Individuals born in high exposed counties are 1.2 and 1.8 percentage-points less likely to reach ages 65 and 75, off a mean of 0.85 and 0.48, respective.

Our sample covers various cohorts with very different life expectancy observed in a limited death window. One concern about this selection is over/under-representation of different cohorts in death records. The CenSoc-extracts of Numident-DMF provides a weighting variable which employs data from Human Mortality Database to correct for this differential representation of cohorts. We use this weight in our regressions and replicate the results in column 8. We observe quite comparable effects to those of column 1.^9^

In column 9, we show that standard errors are quite robust when we employ county-level clustering. Another concern in our analysis is the overlap of the Great Depression with a portion of our sample. Several studies document the potential influence of the Great Depression and the introduction of social programs under the New Deal on short-run and long-run health outcomes (Cutler *et al.*, 2007; Fishback *et al.*, 2007; Modrek *et al.*, 2022; Noghanibehambari and Engelman, 2022; Schmitz and Duque, 2022). To examine the sensitivity of the results, we remove cohorts of 1930s and replicate the results. We find almost identical coefficients compared to column 1.

To further address concerns regarding the potential confounding influence of the Great Depression and contemporaneous New Deal programs, we incorporate additional controls capturing local economic conditions and relief spending. Specifically, we obtain county-level per capita New Deal relief expenditures from Fishback *et al.* (2006) and interact this measure with birth-year fixed effects, allowing the influence of relief spending to evolve flexibly across cohorts. The results, reported in Appendix Table A-9, closely mirror the baseline estimates and indicate that the main findings are not driven by differential exposure to New Deal relief programs.

In a related exercise, we use county-level retail sales per capita in 1929, also from Fishback *et al.* (2006), as a proxy for pre-Depression local economic conditions. We interact this measure with birth-year fixed effects and include these interactions in the baseline specification to account for differential cohort exposure to local economic environments at the onset of the Great Depression. The estimates reported in Appendix Table A-10 remain comparable to the main results.

Throughout the main analysis, we define exposure using terciles of baseline textile employment. We group counties into terciles of baseline textile employment to capture potential non-linearities in exposure intensity while maintaining adequate statistical power. This categorical specification is useful when industrial dependence is highly skewed across geographic units and the number of observations is limited. In Appendix Table A-2, we test the sensitivity of our findings to alternative functional forms of the exposure variable. The results remain robust when using a continuous standardized measure of baseline textile employment (column 1) or when defining exposure using quartiles or quintiles (columns 2–3). Across all specifications, the point estimates exhibit a consistent gradient pattern, with larger negative effects observed in higher quartiles and quintiles of baseline textile employment, mirroring the main results.

Another potential concern is the confounding influence of the 1918–1919 influenza pandemic. Prior research documents long-term adverse effects of in utero exposure to this pandemic on individuals’ later-life health outcomes. To assess the robustness of our findings, we first exclude the 1918–1919 birth cohorts and re-estimate the models. As reported in column (1) of Appendix Table A-3, the coefficients become even larger in magnitude, suggesting that the true effects may, if anything, be understated in the main results. Next, we incorporate city-level and state-level influenza death rates from Beach *et al.* (2022a) and Tycho (2021) to assign influenza mortality rates to counties. We then divide counties into the top two terciles and the bottom tercile of influenza mortality and replicate the analysis in columns (2) and (3) of Appendix Table A-3. Although the point estimates lose statistical significance (likely due to smaller sample sizes), they remain comparable in magnitude to the main results, indicating similar reductions in longevity across both subsamples.

A potential concern is that multiple individuals in the sample may belong to the same family, which could introduce within-household correlation in outcomes. Using household identifiers available in the 1940 census, we identify siblings residing in the same household and construct a restricted sample that retains only one individual per family. Re-estimating the baseline specification using this sample, reported in Appendix Table A-13, yields results that are quantitatively similar to the main estimates.

Finally, to examine whether aggregation of exposure at the county level influences our results, we construct an alternative measure of textile dependence using town-level data. Specifically, we link the full-count censuses from 1900–1940 to town identifiers provided by Berkes *et al.* (2023). We then generate a town-level exposure measure analogous to our baseline county-level measure. We then re-estimate the baseline specification while including town fixed effects. The results, reported in Appendix Table A-11, are similar to the main estimates and exhibit an analogous pattern of effects across subpopulations. The use of town-level exposure raises the question of how treatment assignment differs across geographic aggregation levels and which spatial unit most appropriately captures local labor market shocks. Two considerations motivate our use of county-level exposure in the baseline analysis. First, county-level treatment more closely approximates integrated local labor markets during the early twentieth century. Textile production, employment opportunities, and supporting infrastructure were typically organized across clusters of neighboring towns rather than confined within municipal boundaries. Consequently, plant closures and industrial decline likely affected households through county-wide labor market conditions rather than isolated town-specific shocks. Second, New England towns are geographically small and closely connected, with substantial economic interaction across municipal borders (Bartik, 2024; Boustan *et al.*, 2010; Choi, 2023). Assigning treatment at the town level may therefore treat nearby locations exposed to the same underlying labor market shock as independent units, raising concerns related to spillover effects and spatial dependence when workers reside in one town but work or access services in adjacent towns. From this perspective, county-level exposure provides a measure of shared economic conditions faced by families during early life. To quantify the extent to which treatment classification differs across the two geographic definitions, Appendix Table A-12 reports the distribution of county-level exposure terciles within each town-level exposure group. The table shows substantial concordance between the two measures: a large majority of observations classified in the lowest and highest town-level exposure terciles are assigned to the corresponding county-level terciles, while reassignment across extreme exposure categories remains limited. This pattern indicates that town-level variation primarily reflects within-county heterogeneity rather than fundamentally different treatment assignment. Consistent with this evidence, the estimates reported in Appendix Table A-11 remain highly similar to the baseline results across subsamples. We therefore retain county-level exposure as the primary specification while interpreting town-level estimates as a robustness exercise demonstrating that the main findings are not driven by geographic aggregation.

## 7. Mechanisms

In this section, we first review the theoretical framework for the pathway linking early-life economic conditions to old-age longevity. We then provide several pieces of empirical evidence to support these claims using available data.

The most direct mechanism involves resource deprivation, whereby parental job loss constrains family resources needed for essential health inputs, such as nutrition, healthcare access, and adequate housing. For example, evidence from historical famine episodes shows that severe nutritional deprivation during in utero exposure can generate persistent reductions in health and longevity later in life (Lindeboom *et al.*, 2010; McEniry *et al.*, 2008; Roseboom *et al.*, 2001; Van Abeelen *et al.*, 2012). While the economic shock examined in our setting is considerably less severe than famine conditions, this literature highlights the broader sensitivity of early-life health to adverse resource environments during critical developmental periods. In the context of textile plant closures, reduced household income may have limited access to healthcare and increased vulnerability of infants and children to disease exposure, mechanisms that have been shown to influence long-run health and mortality outcomes (Almond, 2006; Almond *et al.*, 2012; Beach *et al.*, 2016; Case and Paxson, 2009; Cook *et al.*, 2019; Fletcher, 2018). On the other hand, worsening economic conditions in the wake of plant closures might induce stress among the whole community. This is particularly relevant for prenatal development and infant health outcomes but has also been documented to significantly influence later-life outcomes (Álvarez-Aranda *et al.*, 2020; Carlson, 2015; Entringer *et al.*, 2011; Torche, 2011). Aizer *et al.* (2016b) find that elevated stress hormones (cortisol) during pregnancy worsen offspring cognition, health, and educational attainment.

Further, disruptions during critical skill formation periods fundamentally alter an individual’s life-cycle trajectory, as established by the technology of skill formation literature (Cunha *et al.*, 2010; Cunha and Heckman, 2007). During the period studied, formal public social insurance programs were considerably more limited in scope and coverage than those available in many settings today, although families may have relied on alternative informal support mechanisms such as extended family networks or community assistance. In this context, the loss of labor income could nonetheless constrain household investments in children, potentially leading families to withdraw children from school or reduce expenditures on education. Consistent with this mechanism, Mari and Keizer (2021) find that parental job loss during the Great Recession in Ireland is associated with increases in children’s behavioral problems at ages 3 and 5 and reduced verbal ability at age 3, with these effects operating primarily through reductions in parental income and increased maternal negative parenting.

Additionally, economic shocks can operate through persistent behavioral and psychosocial scarring that leads to chronic stress and poor health behaviors. Parental job loss is a major source of family stress, which is often found to reduce the subjective well-being of children well into adulthood (Nikolova and Nikolaev, 2021). The resulting changes in parental mental health, behavior, and family stability can impede a child’s development through chronic exposure to adverse environments (Goodman *et al.*, 2011). Lantz *et al.* (1998) find that individuals who experienced economic hardship during childhood were more prone to engage in risky health behaviors in adulthood, including heavy smoking, drinking, and drug use, which in turn increase the risk of mortality. Van Den Berg *et al.* (2010) find that shocks to local labor demand in early-life are associated with later-life reductions in cognitive function and memory, affecting health management. Mooi-Reci and Wooden (2022) report that children exposed to prolonged parental unemployment suffer much worse mental health in adulthood.

Finally, beyond individual and household-level mechanisms, the decline of the textile industry likely generated collective consequences that may have contributed to reduced longevity through the erosion of local public goods and infrastructure. The sharp contraction in manufacturing employment might have led to a shrinking tax base, potentially constraining the ability of highly exposed counties to maintain or invest in healthcare infrastructure (Choi, 2023). This fiscal decline could have operated through two channels: first, as an early-life exposure, limiting children’s access to adequate sanitation, public health services, and educational infrastructure during critical developmental stages; and second, as a direct, lifelong constraint on non-migrants who remained in communities where hospitals and public works may have deteriorated over time. Historical evidence suggests that programs enhancing local fiscal capacity, such as New Deal relief, were associated with reductions in later-life mortality, and early investments in public health institutions, such as county health departments or water treatment facilities, appear to have yielded substantial long-term health benefits (Cutler and Miller, 2005, 2022; Hoehn-Velasco, 2018, 2021; Modrek *et al.*, 2022). In this context, the decline of the New England textile base may have imposed a negative fiscal shock that plausibly diminished the health capital of exposed cohorts by degrading the broader public environment throughout their lives.

### 1. Economic downturn

A growing literature links in utero and early-life economic conditions to later-life health and mortality through resource- and investment-based channels (Aizer *et al.*, 2016a; Noghanibehambari *et al.*, 2024; Schmitz and Duque, 2022; Van Den Berg *et al.*, 2006, 2009; van den Berg and Gupta, 2015). The changes in employment prospects and county-level economic conditions following the New England textile decline provide a plausible mechanism through which early-life exposure translated into reduced longevity. We examine this channel by regressing several county characteristics on measures of exposure, conditional on county and year fixed effects. As shown in Appendix Table A-4, counties with higher initial dependence on textile manufacturing experienced substantial employment losses after 1920, reflected in a 1.4 percentage-point decline in employment among those in the labor force and a 2.4 percentage-point reduction in the share of workers in the textile industry. These shocks were only partially offset by small increases in agricultural, transportation, and other low-wage sectors, while the overall socioeconomic index declined by about 1.5 units, indicating a broad deterioration in local economic conditions.

These findings align with the extensive literature on the long-term effects of early-life economic shocks, which suggests that adverse labor market conditions during critical developmental periods can have persistent impacts on health and mortality through reduced household income, poorer nutrition, limited access to medical care, and diminished parental investments in children’s education and health (Goodman *et al.*, 2011; Hayward and Gorman, 2004; Hoehn-Velasco, 2021; Hollingsworth *et al.*, 2024; Lindeboom *et al.*, 2010; Smith *et al.*, 2009; Van Den Berg *et al.*, 2006). Overall, the county-level evidence strengthens the interpretation that the long-run mortality effects documented in this paper operate, at least in part, through the observed declines in employment prospects and local economic opportunity during early life.

Further, we observe an increase in the share of whites and a corresponding decline in the share of Blacks (columns 9–10), suggesting that following the economic shock, Black residents in the most affected counties were more likely to migrate. To the extent that average longevity among whites exceeds that of Blacks, this compositional shift likely leads to a downward bias in our estimates, implying that the true effects of early-life exposure to deindustrialization may be even larger. Finally, we find small but statistically significant reductions in fertility (column 11), which may reflect families’ adjustments to worsening local economic conditions through delayed or foregone childbearing.

### 2. Infant mortality

Another potential pathway linking early-life exposure to local economic shocks and later-life mortality is through infant health. A large body of research documents that infant health outcomes are highly sensitive to local economic conditions and household income fluctuations (Almond and Currie, 2011; Baird *et al.*, 2011; Chung *et al.*, 2016; Hoynes *et al.*, 2011, 2015; Lindo, 2011). The deterioration in employment opportunities and local economic activity following deindustrialization could therefore manifest in poorer infant health and higher infant mortality rates. To examine this mechanism, we use county-level natality and mortality data from Bailey *et al.* (2016) and estimate the effects of deindustrialization exposure on infant mortality and birth rates. Given that the natality data cover a limited set of counties beginning in 1915, we focus on comparing counties in the top tercile of baseline textile employment with all other counties, as further disaggregation substantially reduces statistical power. The results, presented in Appendix Table A-5, show that counties with high baseline textile dependence experienced an increase in infant mortality rates of approximately 2.9 per 1,000 live births, off a mean of 80 (column 1), with similar findings when using a log-transformed specification (column 2). However, we do not find statistically significant effects on birth rates per population (columns 3–4).

### 3. Education and socioeconomic standing

Education and general socioeconomic status are among the potential mediators between early-life shocks and old-age outcomes (Almond *et al.*, 2018; Currie, 2009). Further, several studies point to the potential benefits of education and income for longevity (Chetty *et al.*, 2016; Fletcher, 2015; Lleras-Muney, 2005; Meghir *et al.*, 2018; Salm, 2011). The census provides information on education and various measures of socioeconomic standing, which we could use to explore mechanism channels between early-life shocks and later-life longevity. However, many of post-1920 cohorts have not finished their education or not in the labor market by 1940. Therefore, we move to later censuses to examine these potential impacts. One drawback is that the public-use censuses of 1950-onwards do not report county. However, IPUMS extracts de-identify county based on other available geographic variables. We use 1950 and 1960 censuses and the limited de-identified counties to examine the mechanisms.^10^ We limit the sample to individuals aged at least 22 and whose state-of-birth is the same as state-of-residence in the census to mitigate the influence of migrants. We implement regressions similar to equation 1 and report the results in Table 8. Among top-tercile of exposure counties, we observe significant increases in years of schooling. We do not observe an effect among those at the second tercile, a pattern that is consistent with the results of longevity in Table 3. We also observe significant increases in the likelihood of being low educated (columns 2–3) and reductions in having a college education (column 4). We also observe significant and relatively large reductions in socioeconomic measures (columns 5–6). High exposure (top-tercile of 1900 textile) is associated with 2.3 and 2 units reductions in socioeconomic index and occupational educational score, off a mean of 37.1 and 19.4, respectively. Additionally, high exposure is associated with a significant reduction in the probability of having a white-collar occupation of roughly 1.2 percentage points, off a mean of 0.08 (column 7). It is also associated with a reduction of 2.1% in family income (column 8).

**Table 8.** Exploring mechanisms using 1950−1960 census

	Outcome: age at death (months), subsamples:							
	Years of schooling	Years of schooling < 9	Years of schooling < 12	Education: college-more	Socioeconomic index	Occupational education score	White-collar occupation	Log family income
	(1)	(2)	(3)	(4)	(5)	(6)	(7)	(8)
3^rd^ Tercile of 1900 Textile 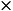 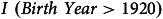	−0.3196***	0.0364***	0.0354***	−0.0354***	−2.4218***	−2.2716***	−0.0115***	−0.0213***
	(0.0473)	(0.0078)	(0.0038)	(0.0038)	(0.2902)	(0.2352)	(0.0026)	(0.0072)
2^nd^ Tercile of 1900 Textile 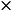 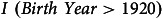	−0.0526*	0.021***	0.0029	−0.0029	0.0369	−0.2853	−0.0015	0.012
	(0.0298)	(0.005)	(0.004)	(0.004)	(0.2453)	(0.2261)	(0.0026)	(0.008)
Observations	255038	255038	255038	255038	198227	197619	255038	238983
R-squared	0.2313	0.1667	0.0283	0.0283	0.0341	0.0385	0.0125	0.1517
Mean DV	7.460	0.294	0.834	0.166	37.848	20.917	0.081	8.684
County FE	✓	✓	✓	✓	✓	✓	✓	✓
Birth year FE	✓	✓	✓	✓	✓	✓	✓	✓
Census year FE	✓	✓	✓	✓	✓	✓	✓	✓
Controls	✓	✓	✓	✓	✓	✓	✓	✓

*Notes*: Standard errors, two-way clustered on county and birth-year, are in parentheses. Controls include individual and county covariates. Individual controls include dummies for race and ethnicity. County controls include average population, the share of population in different age groups, share of population in different race groups, share of immigrants, share of married individuals, average family size, and average occupational income score.

*** *p* < 0.01, * *p* < 0.1.

We can compare these estimates with other studies that examine determinants of mortality to understand what portion of effects could pass through these channels. For instance, Halpern-Manners *et al.* (2020) employs Numident data, implements twin fixed effect strategy, and documents that an additional year of schooling is associated with about 4 months higher age at death. Combining this estimate with column 1 of Table 8, one can deduce a longevity reduction of 1.2 months through decreases in education, which is about 33 percent of the reduced-form of Table 3.

## 8. Discussion

The results suggest that high exposure to deindustrialization have a long-lasting effect among non-migrants and individuals born in rural areas. The estimated effects reveal an ITT effects of about 3.3 months. To understand the economic magnitude of this effect, we compare it with other studies of mortality and other early-life shocks. Fletcher and Noghanibehambari (2023) examine the impacts of college openings on college education and longevity using Numident data. They find treatment-on-treated (TOT) effects of 1 year of additional life for college-educated individuals. Therefore, high exposure to the industrial decline in early-life can offset about 28 percent of benefits of college education for longevity. Aizer *et al.* (2016a) examine the long-term effects of a social program in the early 20^th^ century on old-age mortality. They study Mothers Pension (MP) program which was designed to pay cash benefits to poor single mothers for a period of three years. The payments accounted for about 30–40 percent of pre-transfer maternal income. They show that children of mothers whose applications were accepted for MP live about 12 months additional lives. Therefore, our ITT effects are about 30 percent of the TOT benefits of a relatively large cash transfers which lasted three years. Chetty *et al.* (2016) examine the income-longevity relationship using individual-level tax returns and mortality database over the years 1999–2014 in the US. They find that each additional income percentile (about $8K change from the mean in 2020 dollars) is associated with about 1.9 months higher longevity. Therefore, early-life exposure to the severe deindustrialization of textile industry is equivalent to the longevity effects of a reduction of about $15K in household income.

Although we find considerable heterogeneity in Table 4, these estimates are still ITT effects. One way to convert them into TOT effects is to use the first-stage effects of exposure on textile employment using a similar regression to equation 1 and a county-census-year data. We find that high-exposed counties (top-tercile of 1900 textile) reveal about a 7-percentage-point drop in textile employment. Using the ITT effect of 3.6 and deflating by the first-stage effect of 0.07, a back-of-an-envelope calculation suggests a TOT impact of about 4.2 years among non-urban non-migrants whose fathers lost their job due to deindustrialization.

Life expectancy at age 35 (the minimum age at death in the final sample) in the US increased from about 35.25 years in 1900 to 41.58 years in 1940. Our results suggest that longevity reduction due to a high exposure to the New England textile decline is equivalent to about 4.7 percent of overall life expectancy difference across cohorts in the final sample.

Using the original 1940 census, non-migrant cohorts in high exposure counties born after 1920 count to about 101.6K individuals. Assigning the ITT effects of column 6 of Table 3, one can calculate roughly 30,480 life-years lost due to early-life exposure to deindustrialization.

### 1. Conclusion

Changes in local labor market conditions may have spillover impacts on short-run and long-run health outcomes. The studies that examine the link between economic conditions and health report mixed evidence which differ by the outcome, subpopulation, and setting. While health outcomes of adults, and specifically working age populations, usually deteriorate during economic booms (Ruhm, 2000, 2015, 2016), infants’ and children’s health outcomes, on the other hand, reveal a procyclical behavior (Baird *et al.*, 2011; Page *et al.*, 2019; Waldmann, 1992). An important and policy-relevant question is the extent to which these negative impacts persist. A strand of literature examine this question for a wide range of life-cycle outcomes (Almond *et al.*, 2018; Almond and Currie, 2011). However, little is known about the link between early-life economic shocks and old-age longevity. This article explores this question using a large-scale deindustrialization case: the decline in New England textile industry during the 1920s and 1930s.

We show that worsening demand and increasing competition resulted in large reductions in textile employment in New England post-1920, specifically for counties with higher initial reliance on textile. The effects on migration is small and mainly concentrated among non-urban individuals, a finding consistent with previous studies (Choi, 2023). We then implement event-studies and difference-in-difference regressions to compare old-age longevity of individuals born in counties with higher versus lower exposure to the deindustrialization after 1920s versus before. We find reductions in longevity of about 3.6 months for exposed cohorts who reside in non-urban areas and whose family did not migrate from their county-of-birth until 1940. We also find substantial heterogeneity in the effects with the largest impacts among illiterate fathers, low socioeconomic status fathers, and those whose fathers’ reported occupation is in textile industry. Further, we show that during early adulthood, exposed individuals have lower educational attainment and significantly lower measures of socioeconomic standing.

We should note that this study relates to a period with limited social insurance programs. Although one should exercise caution in interpreting the results for other settings; it has the advantage of helping us better isolate the effects of economic shocks not confounded by health benefits of social programs. The fact that we find long-lasting effects for early-life economic shocks calls for restructuring preexisting policies and introducing new ones with a more focus on families with infants and children. Furthermore, we find that the negative effects are concentrated among non-urban population, those with lower alternatives in the labor market, and non-migrants. Therefore, our results also contain policy suggestions to facilitate migration and occupational mobility during demand shocks, for instance, increased foreign competition after trade policy changes.

## Supporting information

10.1017/dem.2026.10021.sm001 Noghanibehambari and Fletcher supplementary material Noghanibehambari and Fletcher supplementary material

## Footnotes

1
Although the literature to examine this channel in early decades of the 20^th^ century is limited, there is evidence that healthcare access was an important Contributor to health outcomes during this period. Hoehn-Velasco (2018) examines the effects of county health departments on mortality rates during the period of 1908–1933. She finds advantages in increases in access to child-oriented health services through the opening of county health departments for infant mortality rates.

2
Parental literacy variables are constructed using information from the historical censuses of 1900–1930 and are complemented with parental education measures available in the 1940 census for individuals who cannot be linked to earlier censuses. Maternal education indicators are obtained from the 1940 census and include dummies for education below high school, high school completion, college education, and an additional indicator for missing values. The paternal socioeconomic index is similarly derived from the historical censuses of 1900–1930 and supplemented using the corresponding measure reported in the 1940 census for observations not successfully linked to earlier census records.

3
The linkage is based on “ABE fully automated” method based on first name, last name, and age (Abramitzky *et al.* 2012, 2014, 2017).

4
The HRS data also provides links to 1940 census for about 9K observations. Restricting that linked HRS data to cohorts and states of the final sample of this paper leaves less than a few hundred observations in the final sample, while the DMF-Numident results in about 170K individuals.

5
We use ABE exact names methods as the linking rule (Abramitzky *et al.* 2021).

6
Appendix Table A-8 presents detailed summary statistics comparing individuals across migration and urban status. Importantly, nonurban non-migrant individuals do not appear to differ dramatically in death age from other groups, averaging 905.2 months (roughly 75 years), which is nearly identical to nonurban migrants (905.4 months) and only slightly below urban residents. However, nonurban non-migrants were more likely to be white, have lower parental literacy, and had lower maternal education levels relative to migrants.

7
One concern in interpreting the results is that the timing of exposure to deindustrialization is not clearly connected to the period most relevant for shaping later-life longevity. Specifically, our empirical method relies on cross-age comparison (combined with space – time variation in industrialization) rather than extracting age-specific exposure affects. The event study results of Figure 3 suggests that the effects are concentrated among cohorts born after 1920 (versus older cohorts born before 1920), suggesting that exposure during early life is crucial in this setting. This interpretation is supported by a large body of research emphasizing that in utero and early childhood environments have disproportionately large impacts on long-term health and mortality (Almond and Currie 2011; Barker 1994; Scholte *et al.* 2015; Van Den Berg *et al.* 2006, 2011). As elaborated in the mechanism section, early-life economic distress may impair fetal development, nutrition, immunity, and cognitive foundations, all of which shape later-life health trajectories. While our setting does not permit precise identification of the age at which exposure exerts its strongest effects (since all age groups were affected by the economic decline), the effects can be interpreted as the negative impacts of the deindustrialization for in utero and early life exposure net of negative impacts for later childhood exposures.

8
Since this sample is not linked to historical censuses (as we are evaluating the endogenous merging between censuses as well as Numident-DMF data), we use county-of-1940 and county-of-1935 to detect non-migrants. We also use county-of-1940 as a proxy for county-of-birth.

9
In Appendix A, we report supplementary analyses using the CenSoc-DMF which expands mortality coverage from 1975–2005 to 1975–2020. This update captures a greater share of deaths among later-born cohorts and allows us to explore mortality outcomes over a longer horizon. However, we exercise caution in interpreting results from the extended period due to substantial under-coverage in the DMF after 2005. This incompleteness (driven by the exclusion of state-reported deaths from the DMF) results in lower inclusion probabilities, especially after 2015, and varies considerably across states and racial groups. This is the main reason that we focus on the years 1975–2005 (the so-called high mortality coverage period) in the main analysis. However, the supplementary results reported in the appendix suggest that the main estimates are robust to expanding death window coverage.

10
While the final sample of the main analyses has 67 counties, the sample of 1950–1960 census contains 30 identified counties.

## Supplementary material

The supplementary material for this article can be found at https://doi.org/10.1017/dem.2026.10021
