# Supplementary material for: Early-life local labor market conditions and old-age male mortality: evidence from deindustrialization of New England textile sector
Source: J Demogr Economics. 2026 May 14:1–34. Online ahead of print. doi: 10.1017/dem.2026.10021 (PMC13222764; doi:10.1017/dem.2026.10021)
Supplement: Noghanibehambari and Fletcher supplementary material [file S2054089226100212sup001.pdf]

Appendix A

Appendix Table A-1 - Replicating the Main Results Using the Updated DMF Data Covering the Years 1975 – 2020

	Outcome: Age at Death (Months), Subsamples:					
	Full-Sample	Non-Migrants	Migrants	Non-Urban	Urban	Non-Urban Non-Migrants
	(1)	(2)	(3)	(4)	(5)	(6)
3rd Tercile of 1900 Textile × $I(Birth\,Year > 1920)$	.68697<br>(.77797)	-1.13051<br>(1.18243)	1.64855**<br>(.82458)	-1.47697<br>(.98268)	.94709<br>(.77861)	-3.05492*<br>(1.63337)
2nd Tercile of 1900 Textile × $I(Birth\,Year > 1920)$	.52573<br>(.92003)	-.2881<br>(1.15543)	.63685<br>(1.06004)	-1.12055<br>(.91366)	-.38178<br>(.95007)	-.3739<br>(1.54405)
Observations	1076802	420457	656345	430858	645944	176775
R-squared	.45041	.38301	.48729	.46055	.44357	.3941
Mean DV	911.566	912.954	910.678	909.722	912.796	911.171
County FE	✓	✓	✓	✓	✓	✓
Birth Year FE	✓	✓	✓	✓	✓	✓
Controls	✓	✓	✓	✓	✓	✓

Notes. Standard errors, two-way clustered on county and birth-year, are in parentheses. Controls include individual, family, and county covariates. Individual controls include dummies for race and ethnicity. Family controls include dummies for maternal education, paternal literacy, and paternal socioeconomic index. County controls include average population, the share of population in different age groups, share of population in different race groups, share of immigrants, share of married individuals, average family size, and average occupational income score.
*** p<0.01, ** p<0.05, * p<0.1

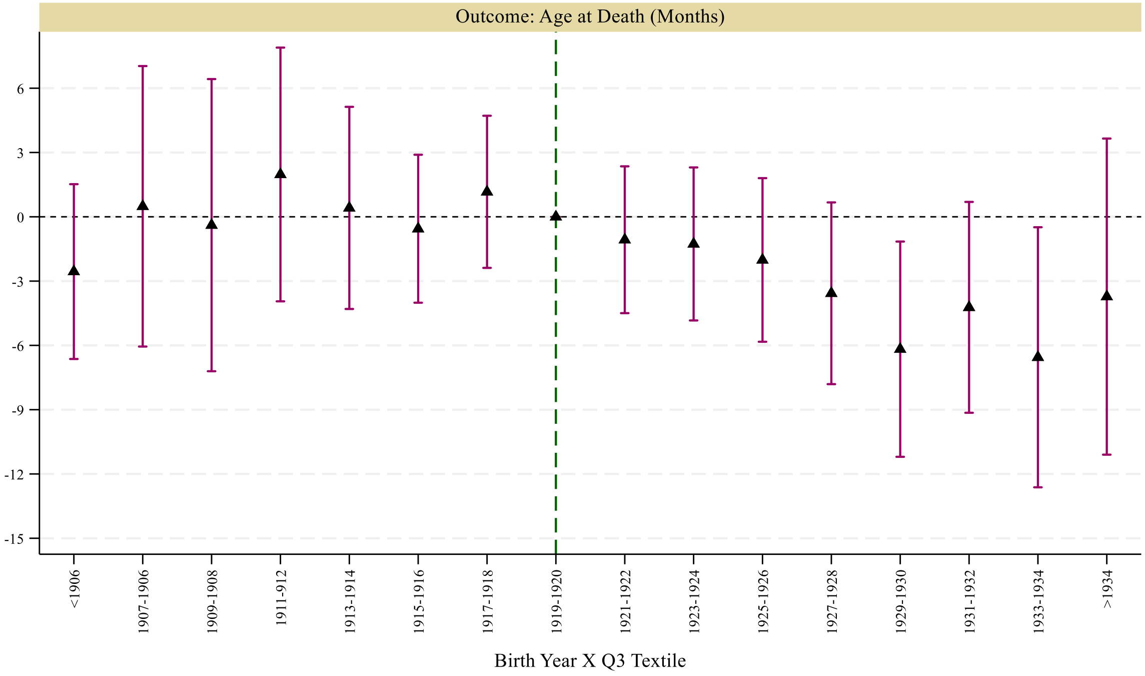

Appendix Figure A-1 - Replicating the Event Study Results Using the Updated DMF Data Covering the Years 1975 – 2020

Notes. Point estimates and 90 percent standard errors are depicted. Standard errors are two-way clustered on county and birth-year. Regressions include county fixed effects, birth year fixed effects, and controls. Controls include individual and county covariates. Individual controls include dummies for race and ethnicity. County controls include average population, the share of population in different age groups, share of population in different race groups, share of immigrants, share of parried individuals, average family size, and average occupational income score.

Appendix Table A-2 - Examining Robustness to Alternative Baseline Exposure

	Outcome: Age at Death (Months)		
	(1)	(2)	(3)
1900 Textile (STD) × $I(Birth\ Year > 1920)$	-2.29518***		
	(.58503)		
4th Quartile of 1900 Textile × $I(Birth\ Year > 1920)$		-5.54032***	
		(2.0105)	
3rd Quartile of 1900 Textile × $I(Birth\ Year > 1920)$		-3.19295*	
		(1.81683)	
2nd Quartile of 1900 Textile × $I(Birth\ Year > 1920)$		-1.44932	
		(1.81905)	
5th Quintile of 1900 Textile × $I(Birth\ Year > 1920)$			-7.37749***
			(2.26537)
4th Quintile of 1900 Textile × $I(Birth\ Year > 1920)$			-5.28421***
			(1.96941)
3rd Quintile of 1900 Textile × $I(Birth\ Year > 1920)$			-2.63942
			(1.91084)
2nd Quintile of 1900 Textile × $I(Birth\ Year > 1920)$			-.65091
			(2.05943)
Observations	166920	166920	166920
R-squared	.46302	.46301	.46304
Mean DV	905.200	905.200	905.200
County FE	✓	✓	✓
Birth Year FE	✓	✓	✓
Controls	✓	✓	✓

Notes. Standard errors, two-way clustered on county and birth-year, are in parentheses. Controls include individual, family, and county covariates. Individual controls include dummies for race and ethnicity. Family controls include dummies for maternal education, paternal literacy, and paternal socioeconomic index. County controls include average population, the share of population in different age groups, share of population in different race groups, share of immigrants, share of married individuals, average family size, and average occupational income score.

*** p<0.01, ** p<0.05, * p<0.1

Appendix Table A-3 - Examining the Influence of 1918 Influenza

	Outcome: Age at Death (Months)		
	Removing Cohorts of 1918 – 1919	Top two Terciles of 1918 Influenza Death Rate	Bottom Terciles of 1918 Influenza Death Rate
	(1)	(2)	(3)
3rd Tercile of 1900 Textile ×	-4.27703***	-3.34032	-2.92524
$I(Birth\ Year > 1920)$	(1.50423)	(3.05141)	(2.17506)
2nd Tercile of 1900 Textile ×	-1.05884	-.88159	-.22115
$I(Birth\ Year > 1920)$	(1.38026)	(3.00916)	(1.68064)
Observations	152316	101855	65065
R-squared	.48513	.46723	.45226
Mean DV	903.275	910.422	897.024
County FE	✓	✓	✓
Birth Year FE	✓	✓	✓
Controls	✓	✓	✓

Notes. Standard errors, two-way clustered on county and birth-year, are in parentheses. Controls include individual, family, and county covariates. Individual controls include dummies for race and ethnicity. Family controls include dummies for maternal education, paternal literacy, and paternal socioeconomic index. County controls include average population, the share of population in different age groups, share of population in different race groups, share of immigrants, share of married individuals, average family size, and average occupational income score.
*** p<0.01, ** p<0.05, * p<0.1

Appendix Table A-4 - Examining Changes in County Characteristics

	Outcomes:										
	Employed, Conditional on Being in the Labor Force	Labor Force Participation	Share Of Workers in Textile Industry	Share Of Workers in Construction Industry	Share Of Workers in Manufacturing Industries, Excluding Textile	Share Of Farmers	Share Of Workers in Transportation	Socioeconomic Index	Share Of Whites	Share Of Blacks	Number Of Under-5 Children in Households
	(1)	(2)	(3)	(4)	(5)	(6)	(7)	(8)	(9)	(10)	(11)
3rd Tercile of 1900 Textile × $I(Year > 1920)$	-.01393***	-.00182	-.02356***	-.00317***	.00322*	.00461*	.00357***	-1.48254***	.00255***	-.00234***	-.00827***
	(.0018)	(.00164)	(.00247)	(.00123)	(.00169)	(.00257)	(.00093)	(.54392)	(.00024)	(.00023)	(.0023)
2nd Tercile of 1900 Textile × $I(Year > 1920)$	.00104	.00098	-.00814***	.00084	-.00039	-.00689***	.00491***	.56702	.00025	-.00005	-.0034
	(.00173)	(.00172)	(.0024)	(.00121)	(.00261)	(.00265)	(.00101)	(.57562)	(.00027)	(.00026)	(.00278)
Observations	2077	2077	2077	2077	2077	2077	2077	2077	2077	2077	2077
R-squared	.9243	.8082	.98645	.87329	.96483	.97297	.94438	.99668	.96364	.95956	.92651
Mean DV	0.938	0.575	0.126	0.065	0.267	0.056	0.070	27.570	0.988	0.011	0.379
County FE	✓	✓	✓	✓	✓	✓	✓	✓	✓	✓	✓
Year FE	✓	✓	✓	✓	✓	✓	✓	✓	✓	✓	✓

Notes. Standard errors, two-way clustered on county and birth-year, are in parentheses. The data covers the years 1900 – 1930.
*** p<0.01, ** p<0.05, * p<0.1

Appendix Table A-5 - Examine the Effects on Infant Mortality And Birth Rate

	Outcomes:			
	Infant Mortality Rate (Per 1,000)	Log Infant Mortality Rate	Births Per Population (Per 1,000)	Log Birth Per Population
	(1)	(2)	(3)	(4)
3rd Tercile of 1900 Textile $\times I(Year > 1920)$	2.9368*	.044**	.4223	.0262
	(1.6771)	(.0196)	(.3916)	(.0209)
Observations	1062	1062	1062	1062
R-squared	.8535	.8488	.9158	.918
Mean DV	80.038	4.353	19.796	2.968
County FE	✓	✓	✓	✓
Birth Year FE	✓	✓	✓	✓
Controls	✓	✓	✓	✓

Notes. Standard errors, two-way clustered on county and birth-year, are in parentheses. Controls include average population, the share of population in different age groups, share of population in different race groups, share of immigrants, share of married individuals, average family size, and average occupational income score.
*** p<0.01, ** p<0.05, * p<0.1

Appendix Table A-6 - Examining the Correlation Between Migration Status and Sociodemographic Characteristics

	Outcomes:						
	White	Black	Female	Father Literate	Father Literate Missing	Mother Literate	Mother Literate Missing
	(1)	(2)	(3)	(4)	(5)	(6)	(7)
Non-Migrant	.00194***	-.00214***	-.01444***	-.01692***	.00028*	-.02515***	.00025
	(.00027)	(.00026)	(.0016)	(.00074)	(.00017)	(.00093)	(.00018)
Observations	1022265	1022265	1022265	1022265	1022265	1022265	1022265
R-squared	.00818	.00786	.01018	.01577	.01884	.02309	.01799
Mean DV	0.992	0.008	0.282	0.944	0.005	0.930	0.005
County FE	✓	✓	✓	✓	✓	✓	✓
Birth Year FE	✓	✓	✓	✓	✓	✓	✓

Notes. Standard errors, two-way clustered on county and birth-year, are in parentheses.
*** p<0.01, ** p<0.05, * p<0.1

Appendix Table A-7 - Examining the Correlation Between Migration/Rural Status and Sociodemographic Characteristics

	Outcomes:						
	White	Black	Female	Father Literate	Father Literate Missing	Mother Literate	Mother Literate Missing
	(1)	(2)	(3)	(4)	(5)	(6)	(7)
Non-Urban Non-Migrant	.00217***	-.00221***	-.01861***	-.0079***	.00126***	-.00571***	.00094***
	(.0003)	(.00029)	(.00172)	(.00096)	(.00033)	(.00105)	(.00031)
Observations	1022265	1022265	1022265	1022265	1022265	1022265	1022265
R-squared	.00815	.0078	.01017	.0147	.01888	.02099	.01801
Mean DV	0.992	0.008	0.282	0.944	0.005	0.930	0.005
County FE	✓	✓	✓	✓	✓	✓	✓
Birth Year FE	✓	✓	✓	✓	✓	✓	✓

Notes. Standard errors, two-way clustered on county and birth-year, are in parentheses.
*** p<0.01, ** p<0.05, * p<0.1

Appendix Table A-8 - Summary Statistics by Migration/Urban Status

	Nonurban non-migrant		Urban non-migrant		Nonurban migrant		Urban migrant	
	Mean	SD	Mean	SD	Mean	SD	Mean	SD
Death Age (Month)	905.2	111.097	908.301	109.895	905.396	122.465	908.39	120.695
Birth Year	1919.296	9.473	1919.165	9.025	1918.833	11.089	1918.544	10.766
Death Year	1994.733	7.657	1994.861	7.594	1994.287	7.885	1994.248	7.904
White	.994	.075	.991	.093	.996	.065	.988	.107
Black	.005	.07	.008	.09	.004	.062	.011	.105
Father Literate	.941	.235	.927	.26	.959	.197	.946	.226
Father Literate Missing	.004	.063	.005	.069	.004	.066	.006	.08
Mother Literate	.934	.249	.897	.303	.958	.201	.929	.256
Mother Literate Missing	.004	.06	.005	.07	.004	.066	.006	.079
Father Socioeconomic Index 1st Quartile	.371	.483	.212	.409	.312	.463	.202	.402
Father Socioeconomic Index 2nd Quartile	.17	.376	.222	.416	.167	.373	.203	.402
Father Socioeconomic Index 3rd Quartile	.166	.372	.225	.418	.162	.368	.2	.4
Father Socioeconomic Index 4th Quartile	.197	.398	.233	.423	.194	.395	.209	.407
Father Socioeconomic Index Missing	.096	.295	.108	.31	.165	.371	.185	.388
Mother Education < High School	.46	.498	.548	.498	.285	.451	.352	.478
Mother Education High School	.257	.437	.205	.404	.206	.404	.177	.382
Mother Education College	.045	.208	.028	.165	.036	.185	.022	.147
Mother Education Missing	.237	.425	.219	.413	.474	.499	.448	.497
Share of Textile Employment	.127	.112	.15	.118	.124	.112	.143	.116
Population	248000.91	237184.57	427628.8	255420.35	255491.73	247582.44	406366.32	268239.23
Share of Population Aged 0-4	.097	.012	.096	.01	.095	.014	.095	.012
Share of Population Aged 5-10	.113	.011	.111	.007	.111	.011	.109	.008
Share of Population Aged 11-18	.139	.011	.138	.008	.139	.011	.138	.008
Share of Population Aged 19-25	.117	.012	.123	.01	.119	.012	.125	.011
Share of Population Aged 26-55	.398	.024	.413	.016	.399	.025	.412	.018
Share of Population Aged 56-more	.136	.031	.119	.023	.136	.032	.122	.026
Share of Females	.502	.013	.507	.009	.502	.013	.507	.01
Share of Families with Children < 5	.386	.068	.376	.042	.379	.072	.37	.053
Share of Whites	.991	.009	.987	.007	.991	.009	.988	.008
Share of Blacks	.008	.008	.012	.007	.008	.008	.012	.008
Share of First-Generation Immigrants	.221	.075	.274	.055	.222	.079	.266	.065
Share of Second-Generation Immigrants	.313	.092	.368	.06	.299	.094	.346	.073
Share of Literate People	.925	.104	.923	.097	.87	.207	.876	.196
Share of Married	.575	.028	.558	.027	.571	.032	.555	.029
Average Family Size	4.263	.297	4.307	.172	4.237	.298	4.273	.197
Average Occupation Income Score	25.049	2.586	26.506	1.39	24.984	2.631	26.232	1.728
	166920		230180		242061		383104	

Appendix Table A-9 - Adding Controls for New Deal Relief Spending

	Outcome: Age at Death (Months), Subsamples:					
	Full-Sample	Non-Migrants	Migrants	Non-Urban	Urban	Non-Urban Non-Migrants
	(1)	(2)	(3)	(4)	(5)	(6)
3rd Tercile of 1900 Textile	-.09	-1.64	1.22	-3.06**	1.02	-5.59***
$\times I(Birth\ Year > 1920)$	(.7)	(1.11)	(.91)	(1.21)	(.94)	(1.93)
2nd Tercile of 1900 Textile	-.47	-1.36	.04	-1.77	-.35	-2.77
$\times I(Birth\ Year > 1920)$	(.8)	(1.28)	(1.04)	(1.18)	(1.19)	(1.9)
Observations	770658	296648	474010	296985	473673	121187
R-squared	.51	.46	.54	.52	.51	.47
Mean DV	907.258	906.893	907.487	905.974	908.064	904.955
County FE	✓	✓	✓	✓	✓	✓
Birth Year FE	✓	✓	✓	✓	✓	✓
Controls	✓	✓	✓	✓	✓	✓

Notes. Standard errors, two-way clustered on county and birth-year, are in parentheses. Controls include individual, family, and county covariates. Individual controls include dummies for race and ethnicity. Family controls include dummies for maternal education, paternal literacy, and paternal socioeconomic index. County controls include average population, the share of population in different age groups, share of population in different race groups, share of immigrants, share of married individuals, average family size, and average occupational income score.
The regressions also include the average county per capita relief spending under New Deal programs interacted with birth-year fixed effects.
*** p<0.01, ** p<0.05, * p<0.1

Appendix Table A-10 - Adding Controls for the Great Depression

	Outcome: Age at Death (Months), Subsamples:					
	Full-Sample	Non-Migrants	Migrants	Non-Urban	Urban	Non-Urban Non-Migrants
	(1)	(2)	(3)	(4)	(5)	(6)
3rd Tercile of 1900 Textile	-.43	-2.5**	1.27	-2.91**	.33	-5.62***
$\times I(Birth\ Year > 1920)$	(.7)	(1.1)	(.92)	(1.18)	(.97)	(1.9)
2nd Tercile of 1900 Textile	-.79	-2.13	.01	-1.75	-1.46	-2.71
$\times I(Birth\ Year > 1920)$	(.82)	(1.3)	(1.07)	(1.18)	(1.21)	(1.88)
Observations	770658	296648	474010	296985	473673	121187
R-squared	.51	.46	.54	.52	.51	.47
Mean DV	907.258	906.893	907.487	905.974	908.064	904.955
County FE	✓	✓	✓	✓	✓	✓
Birth Year FE	✓	✓	✓	✓	✓	✓
Controls	✓	✓	✓	✓	✓	✓

Notes. Standard errors, two-way clustered on county and birth-year, are in parentheses. Controls include individual, family, and county covariates. Individual controls include dummies for race and ethnicity. Family controls include dummies for maternal education, paternal literacy, and paternal socioeconomic index. County controls include average population, the share of population in different age groups, share of population in different race groups, share of immigrants, share of married individuals, average family size, and average occupational income score.
The regressions also include the average retail sale per capita in 1929 interacted with birth-year fixed effects.
*** p<0.01, ** p<0.05, * p<0.1

Appendix Table A-11 - Replicating the Main Results Using Town Level Data

	Outcome: Age at Death (Months), Subsamples:					
	Full-Sample	Non-Migrants	Migrants	Non-Urban	Urban	Non-Urban Non-Migrants
	(1)	(2)	(3)	(4)	(5)	(6)
3rd Tercile of 1900 Textile	-.67	-1.11	-.39	-3.19***	.18	-4.68***
$\times I(Birth\ Year > 1920)$	(.54)	(.78)	(.74)	(.65)	(.87)	(1.22)
2nd Tercile of 1900 Textile	.44	.21	.66	1.05	.3	-.01
$\times I(Birth\ Year > 1920)$	(.54)	(.78)	(.71)	(.74)	(.95)	(1.05)
Observations	1019091	396460	622623	406018	612918	166280
R-squared	.51	.45	.54	.52	.51	.47
Mean DV	907.051	907.011	907.076	905.114	908.317	905.225
Town FE	✓	✓	✓	✓	✓	✓
Birth Year FE	✓	✓	✓	✓	✓	✓
Controls	✓	✓	✓	✓	✓	✓

Notes. Standard errors, two-way clustered on town, are in parentheses. Controls include individual, family, and town covariates. Individual controls include dummies for race and ethnicity. Family controls include dummies for maternal education, paternal literacy, and paternal socioeconomic index. County controls include average population, the share of population in different age groups, share of population in different race groups, share of immigrants, share of married individuals, average family size, and average occupational income score.
*** p<0.01, ** p<0.05, * p<0.1

Appendix Table A-12 – Tercile Concordance Matrix: Distribution of County-Level Textile Exposure Terciles Within Town-Level Exposure Groups

	First Tercile, Town-Level Baseline Textile	Second Tercile, Town-Level Baseline Textile	Third Tercile, Town-Level Baseline Textile
First Tercile, County-Level Baseline Textile	68.5%	35.8%	7.2%
Second Tercile, County-Level Baseline Textile	24.2%	38.5%	35.1%
Third Tercile, County-Level Baseline Textile	7.3%	25.7%	57.7%
Total	100%	100%	100%

Appendix Table A-13 - Replicating the Main Results Excluding Siblings

	Outcome: Age at Death (Months), Subsamples:					
	Full-Sample	Non-Migrants	Migrants	Non-Urban	Urban	Non-Urban Non-Migrants
	(1)	(2)	(3)	(4)	(5)	(6)
3rd Tercile of 1900 Textile	-.19	-1.65	.86	-2.1*	.99	-3.56*
$\times I(Birth\ Year > 1920)$	(.72)	(1.12)	(.89)	(1.18)	(1)	(1.96)
2nd Tercile of 1900 Textile	-.34	-.79	.01	-1.38	.41	-.62
$\times I(Birth\ Year > 1920)$	(.72)	(1.16)	(.89)	(1.1)	(1.05)	(1.86)
Observations	814235	289389	524846	327536	486699	122664
R-squared	.52	.47	.54	.53	.51	.48
Mean DV	915.398	914.648	915.812	914.083	916.283	913.508
Town FE	✓	✓	✓	✓	✓	✓
Birth Year FE	✓	✓	✓	✓	✓	✓
Controls	✓	✓	✓	✓	✓	✓

Notes. Standard errors, two-way clustered on town, are in parentheses. Controls include individual, family, and town covariates. Individual controls include dummies for race and ethnicity. Family controls include dummies for maternal education, paternal literacy, and paternal socioeconomic index. County controls include average population, the share of population in different age groups, share of population in different race groups, share of immigrants, share of married individuals, average family size, and average occupational income score.
*** p<0.01, ** p<0.05, * p<0.1
